# Advances in plant male sterility for hybrid seed production: an overview of conditional nuclear male sterile lines and biotechnology-based male sterile systems

**DOI:** 10.3389/fpls.2025.1540693

**Published:** 2025-02-05

**Authors:** Naresh Vasupalli, Kanakachari Mogilicherla, Vahab Shaik, K. R. S. Sambasiva Rao, Shripad R. Bhat, Xinchun Lin

**Affiliations:** ^1^ State Key Laboratory of Subtropical Silviculture, Zhejiang A & F University, Lin’an, Hangzhou, Zhejiang, China; ^2^ Bamboo Industry Institute, Zhejiang A & F University, Lin’an, Hangzhou, Zhejiang, China; ^3^ Department of Biotechnology, ICAR-Indian Institute of Rice Research (IIRR), Rajendranagar, Hyderabad, India; ^4^ Faculty of Forestry and Wood Sciences, Czech University of Life Sciences, Prague, Prague, Czechia; ^5^ Department of Pharmacy, Mangalayatan University-Jabalpur, Jabalpur, Madhya Pradesh, India; ^6^ ICAR-National Institute for Plant Biotechnology, New Delhi, India

**Keywords:** genic male sterility (GMS), biotechnology-based male sterile (BBMS) systems, environment-sensitive genic male sterility (EGMS), long non-coding RNAs (lncRNAs), phased secondary small interfering RNAs (phasiRNAs)

## Abstract

Male sterility forms the foundation of hybrid seed production technology in field crops. A variety of genetically controlled male sterility/fertility systems starting with cytoplasmic male sterility (CMS), genic male sterility (GMS) including conditional male sterility and transgenic-based male sterility have been developed and deployed for heterosis breeding over the past century. Here we review environment-sensitive genic male sterility (EGMS) and biotechnology-based male sterility systems and describe the underlying molecular mechanisms. Advances in crop genomics and discovery of a large number of nuclear genes governing anther/pollen development, which are shared across species, are helping design diverse types of male sterile lines suitable for different crop species and situations. In particular, gene editing offers quick and easy route to develop novel male sterility systems for hybrid seed production. We discuss the advantages and challenges of biotechnology-based male sterility systems and present alternative strategies to address concerns of transgenics. Finally, we propose development of functional male sterility systems based on pollen competition as the future area that holds great promise for heterosis breeding.

## Introduction

Plant male sterility is a condition where the plant fails to produce functional pollen. This failure could be due to defects in anther specification and differentiation, abnormal microsporogenesis and pollen development, non-dehiscent anthers, or the inability of pollen to germinate and fertilize the female gametes (Pei et al., 2024). Male-sterile female lines form the foundation of large-scale hybrid seed production in various crop species. Such hybrids with superior agronomic traits such as higher yield, enhanced disease resistance and stress tolerance have greatly contributed to food, feed and nutritional security (Kim and Zhang, 2018). To date, male sterility has been reported in ~617 plant species, including crops like rice, wheat, Indian mustard, cotton, soybean, and Sorghum (Chen and Liu, 2014; Vasupalli et al., 2021; Ren et al., 2022) and tree species like bamboo (De Souza et al., 2020), olive (Besnard et al., 2000), rubber (Shearman et al., 2014). Besides its utility in F1 seed production, male sterility might be an essential feature of plant evolution and adaptation (Budar and Pelletier, 2001).

**Figure 1:**
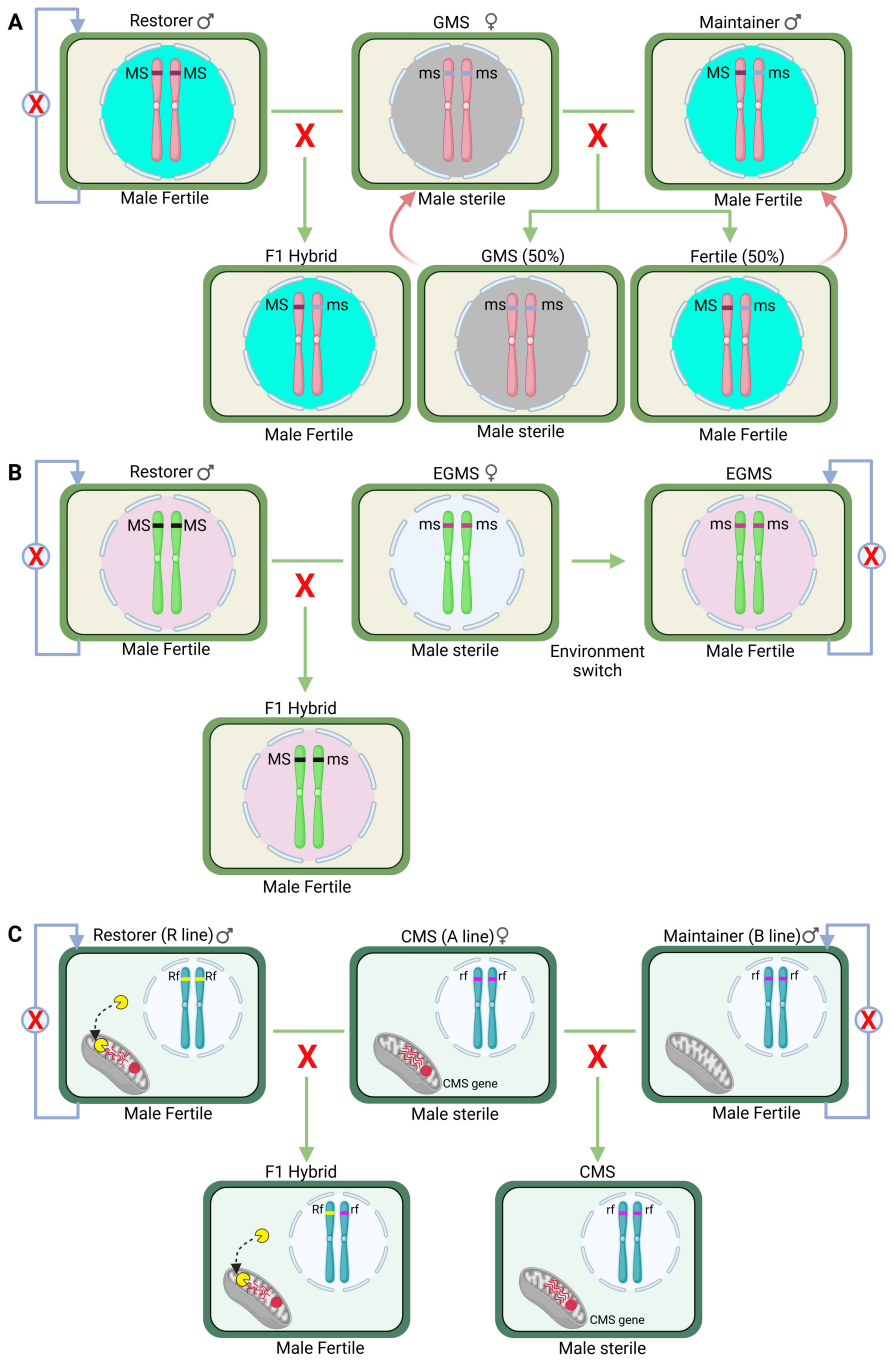
Hybrid breeding using two-line genic or environmental genic male sterility system. **(A)** Genic male sterility (GMS) system comprises of homozygous (ms/ms) male sterile line, hemizygous (Ms/ms) male fertile maintainer line and homozygous (Ms/Ms) wild type line. A cross between the male sterile and the maintainer line yields 50% male sterile and 50% male fertile progenies, from which male sterile plants are used for hybrid seed production by crossing with wild type (Ms/Ms) parent. The other 50% male fertile plants serve as the maintainer line for the next cycle of multiplication of the male sterile line. **(B)** In the EGMS system, the male sterile line (ms/ms) is multiplied by growing under conditions where it becomes male fertile. For hybrid seed production, the male sterile line is raised under conditions favoring male sterility and crossed with wild type (Ms/Ms) male fertile line. **(C)** The three-line cytoplasmic male sterility (CMS) system comprises of the male-sterility-inducing line (A line), the maintainer line (B line) and the restorer line (R line). The A line has CMS-inducing mitochondrial genome but lacks the nuclear restorer gene. The B line is isonuclear to the A line, and has normal mitochondria. The R line carries the nuclear Rf gene and is the male parent of the hybrid. A cross between A X B lines generates all male sterile progeny that could be used either to produce hybrid or to multiply the male steriles. A cross between the A X R lines generates the fertile F1 hybrid seeds.

Various kinds of male sterile lines have been identified/developed to date, such as cytoplasmic male sterility (CMS), genic male sterility (GMS), Environment-sensitive GMS (EGMS), and biotechnology-based male sterile (BBMS) lines. CMS is a maternally inherited trait governed by the mitochondrial genome and rescued by the nuclear genes (Vasupalli et al., 2016). Although CMS has been used commercially in many crops, lack of stable restorer genes and cytoplasmic penalty have severely restricted the expansion of CMS to new crops (Kumar et al., 2012; Chamola et al., 2013). GMS, which is controlled by only nuclear genes, can overcome these disadvantages (Wan et al., 2019). However, the primary disadvantage is that pure male sterile stocks cannot be perpetuated (Anjani, 2005). EGMS is a conditional male sterile system where male sterility/fertility is influenced by environmental conditions such as photoperiod, temperature and humidity. Thus, by growing plants under appropriate environmental conditions, the same stock can be used as male sterile or fertile to produce F1 hybrid seeds or for maintenance, respectively (Peng et al., 2023) (**Figure 1**). Therefore, EGMS systems are preferred for hybrid seed production and widely adopted especially in China. Further, the discovery of GMS genes and advancements in genetic transformation/gene editing technologies have led to the development of BBMS lines (Wu et al., 2016). In this review, we present a brief introduction to GMS and provide in-depth details of molecular mechanisms of EGMS. Further, we also provide recent developments in BBMS systems for hybrid seed production in crop plants.

## Genic male sterility

**Figure 2:**
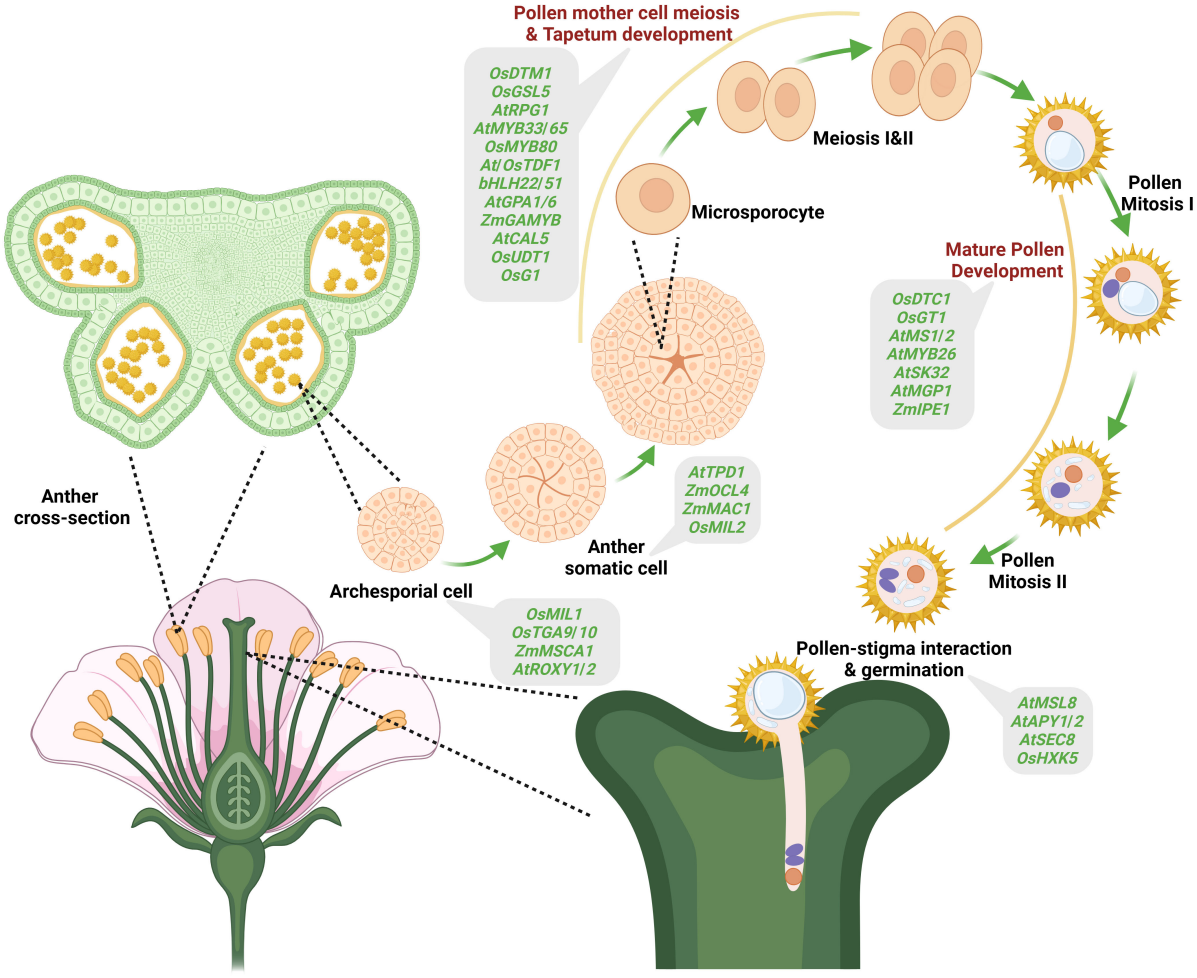
Genes causing male sterility identified in different plant species and their expression at various stages of pollen development. Different stages of pollen development indicated include specification of the archesporial cell, anther somatic cell, pollen mother cell, meiosis and tapetum development (microsporocyte, Meiosis I & II), pollen maturation (pollen mitosis I & II) and pollen-stigma interaction and pollen germination. Various male-sterility-causing genes identified at different stages of pollen development are shown in the ash color boxes.

GMS controlled by nuclear genes is widely reported in plants, which is not surprising considering that anther/pollen differentiation and development involve the interplay of many genes (Cheng et al., 2020). Anther/pollen development comprises different phases viz., archesporial cell specification, somatic differentiation, pollen mother cell meiosis, tapetum development and mature pollen development, and involves cell division, differentiation, development, degradation, and maturation (**Figure 2**). Anther/pollen development by far needs more genes than any other plant organ, and mutations of these genes often lead to complete or partial male sterility (Marchant and Walbot, 2022). Archesporial cells are the premeiotic cells that develop into the plant germline cells, which ultimately develop into gametes. Therefore, defects in archesporial cell development lead to genic male sterility (Zhou et al., 2017). The genes reported to cause GMS in archesporial cell development are *OsMIL1* (Hong et al., 2012b), *ZmMSCA1* (Chaubal et al., 2003), *AtROXY1/2* (Xing and Zachgo, 2008) and *AtTGA9/10* (Murmu et al., 2010). After the archesporial cells specification, somatic cell differentiation takes place. During somatic cell division, many genes are reported to be involved in causing GMS. For example, *AtEMS1/EXS* (Zhao et al., 2002), *AtTPD1* (Yang et al., 2005), *OsMSP1* (Nonomura et al., 2003), *OsMIL2* (Hong et al., 2012a), *OsTIP2/bHLH142* (Fu et al., 2014), *ZmMAC1* (Wang et al., 2012), *ZmOCL4* (Vernoud et al., 2009), and *ZmMs23* (Nan et al., 2017). The four somatic layers, epidermis, endothecium, middle layer and tapetum, are formed from the periclinal somatic cell division. The tapetum is the innermost layer that encircles the developing pollen mother cell and provides the nutrients required for pollen development and also supplies important proteins that coat the surface of pollen grains. Thus, any defect in the development of the tapetum layer causes GMS. Further, tapetum differentiation and meiosis occur simultaneously during anther development. Some of the genes reported that cause defects in tapetum development and pollen mother cell meiosis are *OsTDF1* (Cai et al., 2015), *OsUDT1* (Jung et al., 2005), *ZmMS32* (Moon et al., 2013a), *AtDYT1* (Zhang et al., 2006), *AtMYB33/65* (Millar and Gubler, 2005), *OsGAMYB* (Liu et al., 2010b), *AtNEF1* (Ariizumi et al., 2004) etc. Besides, callose deposition on the newly formed tetrad microspores and timely callose degradation to release the tetrad microspores are crucial in pollen development. Mutations in genes associated with callose metabolism such as *OsDMD1* (Ren et al., 2020), *OsGSL1, OsGSL5* (Enns et al., 2005), *OsLecRK5* (Wang et al., 2020), *OsG1* (Wan et al., 2011), *AtCDM1* (Lu et al., 2014), *AtCalS5* (Dong et al., 2005) have been shown to cause GMS. Further, genes acting at later stages of pollen development, namely, *AtMS1/2* (Ito and Shinozaki, 2002; Chen et al., 2011a), *AtMYB26* (Steiner-Lange et al., 2003), *AtMGP1* (Li et al., 2010)*, AtSK32* (Dong et al., 2015), *OsGT1* (Moon et al., 2013b), *OsDTC1* (Yi et al., 2016), and *ZmIPE1* (Chen et al., 2017) have been identified whose loss-of-function lead to male sterility. Post anthesis, successful pollen germination on the stigma involves crucial steps such as adhesion, hydration, germination and tube growth. A few genes, such as *AtMSL8* (Hamilton and Haswell, 2017), *AtAPY1/2* (Wolf et al., 2007), *AtSEC8* (Cole et al., 2005), and *OsHXK5* (Lee et al., 2020), are reported to be involved in mature pollen interaction with stigma and germination, and mutations in these genes result in male sterility.

Unlike CMS, GMS requires only two lines for hybrid seed production (**Figure 1**). Since all normal lines can serve as restorers, GMS permits the testing of a vast pool of germplasm for the exploitation of heterosis. Besides, there is no cytoplasmic effect. The main hindrance, however, is that a complete male sterile population cannot be obtained, and male sterile plants have to be isolated from a 1:1 mixture of sterile and fertile progeny. Further, as in CMS, two separate isolated fields are needed one each for hybrid seed production and multiplication of male steriles. Nevertheless, GMS has been used for hybrid seed production in some crops such as cotton, cowpea, and vegetable Brassica. To circumvent the problem of isolating male steriles from mixture, conditional male sterile lines were identified which could produce fertile pollen under permissible conditions thereby giving rise to all male sterile offspring. Such conditional male sterile systems form the basis of Environment-sensitive GMS (EGMS) systems, and their molecular mechanisms are discussed below.

## Environment-sensitive GMS

Genes regulating anther development which respond to environmental stimuli will express male sterility under certain environment and are therefore referred to as EGMS (Fan and Zhang, 2018). EGMS was first identified in rice Nongkeng58S (NK58S) by Shi in 1973 (Peng et al., 2023). So far, four types of EGMS responding to photoperiod, temperature, nitrogen status or humidity have been described (Peng et al., 2023). Generally, photosensitive GMS (PGMS) is characterized by male sterility under long days and male fertility under short days. In contrast, ‘reverse PGMS’ is male sterile during short days and male fertile during long days. Thermo-sensitive GMS (TGMS) is characterized by male sterility under high temperature (exceeding a threshold temperature) and male fertility under low temperatures (below a critical sterility-inducing temperature). On the other hand, ‘reverse TGMS’ exhibits the opposite phenotype. Male sterility caused by nitrogen deficiency is known as nitrogen-sensitive genic male sterility (NGMS), where male fertility is observed under conditions of ample nitrogen supply. Additionally, male sterility under low humidity conditions and male fertility under high humidity conditions is referred to as humidity-sensitive genic male sterility (HGMS). Thus, EGMS behaves as male sterile or male fertile under different conditions and, therefore, could be easily maintained by selfing and used for hybrid seed production under appropriate conditions. Hence, EGMS-based pollination control systems are called two-line systems.

EGMS two-line hybrid system (**Figure 1**) offers distinct advantages over CMS or GMS, which include ease of maintenance of male steriles, ready availability of restorers, and male sterility being recessive, the F1s will be fertile under all conditions (Chen et al., 2023; Peng et al., 2023). These features allow rapid improvement of parental lines for quality, stress tolerance etc. However, two-line systems have limitations, such as the need for different locations with suitable environmental conditions, and vulnerability to sudden fluctuations in weather patterns. Further, different environmental conditions may also have adverse effects, such as reduced plant vigor, low yield of hybrid or parental seeds (Chen et al., 2023). Despite these limitations, two-line hybrids have been particularly exploited in China for breeding hybrid rice and contributed significantly to enhance rice production and productivity.

## EGMS genes in different crop species

EGMS has been reported in numerous plant species, such as Arabidopsis (P/TGMS Acos5-2, TGMS Res1 Rpg1, HGMS Cer1-1, Cer1-m) (Aarts et al., 1995; Zhang et al., 2020a; Zhu et al., 2020), rice (PGMS NK58S, TGMS AnS-1, Zhu1S, HGMS *hms1*) (Zhou et al., 2014; Fan et al., 2016; Chen et al., 2020; Wu et al., 2022), maize (TGMS TMS5, MAGO^KD^) (Li et al., 2017; Lee et al., 2021), soybean (rPGMS MS3‐KO) (Hou et al., 2022), wheat (TGMS YanZhan 4110S, P/TGMS K78S and K456S) (Li et al., 2020; Yang et al., 2021), millet (P/TGMS A2) (Wei et al., 2021), tomato (TGMS Da107) (Yu et al., 2015), sorghum (TGMS Ji 130A) (Ma et al., 2012), rapeseed (TGMS TE5A) (Yan et al., 2016), cotton (PGMS CCRI9106) (Zhang et al., 2020b) and barley (rTGMS HvMS1OEx) (Fernandez-Gomez et al., 2020) (**Table 1**). The EGMS genes were identified by fine mapping, map-based cloning, or transcriptome analysis. At least 18 TGMS, five rTGMS, six PGMS, three rPGMS, three P/TGMS, one NGMS and four HGMS genes have been identified in rice (**Table 1**). Similarly, in other plant species, such as Arabidopsis, maize, wheat, soybean, barley, millet and rapeseed, numerous EGMS genes have been identified. The characterization of EGMS genes not only helps devise two-line hybrid systems but also provides a better understanding of plant-environment interactions influencing male reproductive development (Liu et al., 2023). Molecular analyses of various EGMS lines have revealed differential gene regulation operating at different levels such as transcription, translation or post translation, and involving non-coding RNA as responsible for male sterility/fertility transition. Here we focus on the molecular mechanisms of various EGMS systems.

**Table 1:** Details of EGMS genes identified in various crops.

Species	Type of EGMS	EGMS Line	Gene name	Gene ID	Coding product	Pathway	Reference
Arabidopsis	P/TGMS	Acos5-2	*ACOS5*	At1g62940	Acyl-CoA synthetase 5	Pollen exine formation	Zhang et al. (2020a); Zhu et al. (2020)
		Res Rvms-2	*RVMS-1/RVMS*	At4g10950	GDSL lipase/hydrolase	Pollen nexine formation	Zhang et al. (2020a); Zhu et al. (2020)
		Cals5-2, Clas5-6, Res1 Clas5-6	*CalS5*	At2g13680	Callose synthase 5	Pollen exine formation	Zhang et al. (2020a); Zhu et al. (2020)
		Cyp703a2-1, Res1 Cyp703a2-1	*CYP703A2*	At1G01280	Cytochrome P450 703A2	Pollen exine formation	Zhang et al. (2020a); Zhu et al. (2020)
		Npu-2	*NPU*	At3g51610	ATP-dependent helicase/deoxyribonuclease subunit B	Pollen primexine deposition	Zhang et al. (2020a)
	HGMS	Cer1-1, Cer1-m	*CER1*	At1G02205	Acyl‐CoA synthetase	Pollen coat function	Koornneef et al. (1989); Aarts et al. (1995)
		Cer3-8, Cer3-9, Cer3-8m, Cer3-9m	*CER3*	At5G57800	Acyl‐CoA synthetase	Pollen coat function	Koornneef et al. (1989); Xu et al. (2020)
		Cer6-1, Cer6-2	*CER6/CUT1*	At1G68530	Acyl‐CoA synthetase	Pollen coat function	Koornneef et al. (1989); Fiebig et al. (2000)
		Lacs4-1, Lacs4-2	*CER8, LACS4*	At2G47240, At4g23850	Acyl‐CoA synthetase	Pollen coat function	Koornneef et al. (1989); Jessen et al. (2011)
		Cer10-m	*CER10*	At3G55360	Acyl‐CoA synthetase	Pollen coat function	Koornneef et al. (1989)
		Fkp1-1	*FKP*	At4G11820	3‐hydroxy‐3‐methylglutarylcoenzyme A synthase	Pollen wall synthesis process	Ishiguro et al. (2010)
	TGMS	Res1, Res3, Rpg1	*RPG1/SWEET8*	At5g40260	Ruptured pollen grain1/SWEET8	Pollen nexine formation	Zhu et al. (2020); Wang et al. (2022); Zhang et al. (2022a)
		Abcg26-1, Res1 Abcg26-1	*ABCG26*	At3G13220	ATP‐binding cassette transporter G26	Pollen exine formation	Zhu et al. (2020)
		Tms1-1	*TMS1*	At3g08970	HSP40	Growth of pollen tubes, unfolded protein response of ER	Yang et al. (2009)
		Ire1a-2 Ire1b-4	*IRE1A IRE1B*	At2g17520, At5g24360	Endoribonuclease/protein kinase	Pollen coat formation, unfolded protein response of ER	Deng et al. (2016)
		Atsec62 (14-6), Atsec62(27-2)	*AtSec62*	At3g20920	Translocation protein	Protein translocation and secretion	Mitterreiter et al. (2020)
		PEAMT-t365	*PEAMT*	At3G18000	S-adenosyl-l-methionine: phosphoethanolamine N‐Methyltransferase	Signal transduction processes	Mou et al. (2002)
		Pub4-1, Pub4-2, Pub4-3	*AtPUB4*	At2G23140	E3 ubiquitin ligase	Protein degradation/Posttranslational regulation	Wang et al. (2013)
		Coi1-2, Coi1-8	*COI1*	LOC9315901	F box protein	Protein degradation/Posttranslational regulation	Yan et al. (2013); Huang et al. (2014)
		Ice1-2	*ICE1*	At3G26744	MYC-like bHLH transcription factor	Anther dehiscence, transcriptional regulation	Wei et al. (2018)
		Myb33 myb65-2m	*MYB33 & MYB65*	At5G06100, At3G11440	R2R3 MYB transcription factor	Tapetum PCD, transcriptional regulation	Millar and Gubler (2005)
		Bzip60-m	*BZIP60*	At1G42990	bZIP transcription factor	Transcriptional regulation	Iwata et al. (2008)
Maize	TGMS	Dcl5-1 mutant	*DCL5*	LOC103643440	Dicer‐like 5	PhasiRNAs production	Teng et al. (2020)
		ZmTMS5 mutant	*TMS5*	LOC100285786	Rnase ZS1	mRNA decay	Li et al. (2017)
		MAGO^KD^ (line T02878_006)	*MAGO1*, *MAGO2*	Zm00001d007786, Zm00001d013063	MALE-ASSOCIATED ARGONAUTE	Pre-meiotic phasiRNA pathways	Lee et al. (2021)
		Mei025 mutant	*INVAN6*	Zm00001d015094	Alkaline/neutral invertase	Sugar accumulation, metabolism, and signaling	Huang et al. (2022)
		Qiong68ms	*Zmtms3*	Unknown	Unknown	Unknown	Tang et al. (2006)
	TGSI	HE97	*TGSI1*	Unknown	Unknown	Unknown	Lin et al. (2009)
Rice	TGMS	Ugp1-OX, Ugp1-AS, Ugp1-RI	*UGP1*	Os09g0553200	UDP‐Glucose Pyrophosphorylase1	RNA processing	Chen et al. (2007)
		Annong S-1	*TMS5*	Os02g0214300	RNase ZS1	RNA processing	Liang-Bi et al (1994); Zhou et al. (2014)
		TMS10	*TMS10-TMS10L*	Os03g49620	LRR–RLK	Signal transduction processes	Yu et al. (2017)
		Zhu 1S	*TMS9‐1/OSMS1*	Os09g0449000	PHD finger protein	Protein location and transcriptional regulation	Sheng et al. (2013); Qi et al. (2014); Wu et al. (2022)
		Ostms18	*OsNP1*/*OsTMS18*	Os10g38050	GMC oxidoreductase	Pollen exine formation	Zhang et al. (2022b)
		Ostms15	*TMS5*	Os01g68870	LRR-RLK protein (MSP1)	Tapetum development	Han et al. (2023)
		ID24 & SA2	*TGMS*	Unknown	Unknown	Unknown	Reddy et al. (2000)
		5460S	*TGMS1*	Unknown	Unknown	Unknown	Wang et al. (1995)
		Norin PL12, tms2	*ORMDL*/*TMS2*	Os07g26940	Orosomucoid	Sphingolipid homeostasis, PCD	Yamaguchi et al. (1997); Chueasiri et al. (2014)
		IR32364TGMS	*TMS3*(t)	Unknown	Unknown	Unknown	Subudhi et al. (1997)
		TGMS-VN1	*TMS4*(t)	Unknown	Unknown	Unknown	Dong et al. (2000)
		Sokcho-MS	*TMS6*	Unknown	Unknown	Unknown	Lee et al. (2005)
		F 61	*TMS8*	Unknown	Unknown	Unknown	Hussain et al. (2011)
		Hengnong S-1	*TMS9‐1*	Unknown	Unknown	Unknown	Qi et al. (2014)
		Oshsp60-3b mutant	*HSP60-3B*	Os10g32550	Heat Shock Protein 60-3B	Starch granule biogenesis, reactive oxygen species (ROS) levels	Lin et al. (2023)
		OE-OsAL5	*OsAL5*	Os05g34640	Alfin like	TMS5 expression	Wen et al. (2021)
	rTGMS	Osago1d-1; Osago1d-2 & Osago1d-3	*AGO1d*	Os06g0729300	Argonaute protein	PhasiRNAs production	Shi et al. (2022); Si et al. (2023)
		J207S	*RTMS1*	Unknown	Unknown	Unknown	Jia et al. (2001)
		YannongS (YnS)	*RTMS10*	Unknown	Unknown	Unknown	Jin-Long et al (2022)
		G20S, Jing226	*TMS6*(t)	Unknown	Unknown	Unknown	Liu et al. (2010a)
		Sterile s44 mutant and Fertile s44 mutant	*OsOAT*	Os03g44150	Ornithine δ-aminotransferase	Cold tolerance	Yan et al. (2023)
	rPGMS	CSA	*CSA*	Os01g0274800	R2R3 MYB transcription factor	Sugar distribution, transcriptional regulation	Zhang et al. (2010); Zhang et al. (2013)
		YiD1S	*RPMS1*, *RPMS2*	Unknown	Unknown	Unknown	Peng et al. (2008)
	PGMS	CSA	*CSA2*	Os05g0490600	R2R3 MYB transcription factor	Sugar distribution, transcriptional regulation	Wang et al. (2021)
		NK58S	*PMS1*	AK242308	lncRNA	lncRNA regulation	Fan et al. (2016);
		NK58S	*PMS3*	AK111270	lncRNA	lncRNA regulation	(Mei et al., 1999); Ding et al. (2012a); Ding et al. (2012b)
		Antisense-OsPDCD5	*OsPDCD5*	AY327105	Programmed cell death 5 protein	Tapetum PCD	Wang et al. (2010)
		OSMYOXIB promoter-GFP transgenic	*OsMYOXIB*	Os02g0816900	Myosin XI B	Nutrition transport, protein location	Jiang et al. (2007)
		HJX74	*S23*	Unknown	Unknown	Unknown	Fang et al. (2019)
		Mian 9S	*PMS4*	Unknown	Unknown	Unknown	Huang et al. (2008)
	P/TGMS	NK58S & PA64S	*P/TMS12‐1* (*PMS3*), Osa-smR5864w	Os12g0545900	lncRNA, smR5864	lncRNA regulation and smRNA regulation	Zhou et al. (2012)
	HGMS	ZH11-hms1-m	*HMS1*, *HMS1l*	Os03g0220100- Os01g0150000	3‐ketoacyl‐CoA synthase 6, Very‐long‐chain enoyl‐CoA reductase	Pollen coat function	Chen et al. (2020)
		E157	*OsOSC12*/*OsPTS1*	Os08g0223900	Bicyclic triterpene synthase	Pollen coat function	Xue et al. (2018)
		*OsCER1*Cas	*OsCER1*/*OsGL1‐4*	Os02g0621300	Acyl‐CoA synthetase	Pollen coat function	Ni et al. (2021)
	NGMS	etfβ	*ETFβ*	Os04g0182800	Electron‐transporting flavoprotein β subunit	Metabolism of branched‐chain amino acids	Yang et al. (2022)
Soybean	rPGMS	MS3‐KO	*MS3*	GLYMA_02G107600	PHD finger transcription factor	Transcriptional regulation	Hou et al. (2022)
Wheat	P/TGMS	K78S and K456S	Unnamed	Unknown	Unknown	Unknown	Li et al. (2020)
		C412S	Unnamed	Unknown	Adenine phosphoribosyl-transferase	Unknown	Zhang et al. (2009)
		BS20	Unnamed	Unknown	Unknown	Unknown	Li et al. (2006)
		337S	*WPTMS1*, *WPTMS2*, *WPTMS3*	Unknown	Unknown	Unknown	Guo et al. (2006); Chen et al. (2011b)
		BS210	Unnamed	Unknown	Unknown	Unknown	Liping et al. (2009)
	TGMS	BS20-T	*tmsBS20T*	Unknown	Unknown	Unknown	Ru et al. (2014)
		KTP116A	*RFV1*, *RFV2*	Unknown	Unknown	Unknown	Song et al. (2013)
		BS366	Unnamed	Unknown	Unknown	Unknown	Liu et al. (2021)
		YanZhan 4110S	*TaMut11* and *TaSF3*	Unknown	WD domain/G-beta repeat protein and LIM domain protein	Pollen development and fertility conversion	Yang et al. (2021)
Barley	rTGMS	HvMS1OEx	*HvMS1*	LOC123121697	PHD finger protein	Transcriptional regulation	Fernandez-Gomez et al. (2020)
Cotton	TGMS	TGMS line 1-1	Unnamed	Unknown	Unknown	Unknown	Palve et al. (2011)
	PGMS	CCRI9106	Unnamed	Unknown	Unknown	Unknown	Zhang et al. (2020b); Ma et al. (2013)
Millet	P/TGMS	A2	*qSiMS6.1*	Millet_GLEAN_10020454	Tetratricopeptide repeat (TPR)-like superfamily protein	Male-sterility	Wei et al. (2021)
Rapeseed	rTGMS	Huiyou50S	Unnamed	Unknown	Unknown	Unknown	Yu et al. (2016)
Sorghum	TGMS	Ji 130A	Unnamed	Unknown	Unknown	Unknown	Ma et al. (2012)
Tomato	TGMS	San Marzano	*VMS*	Unknown	Unknown	Unknown	Rick and Boynton (1967)
		Da107	Unnamed	Unknown	Unknown	Unknown	Yu et al. (2015)
	P/TGMS	7B-1	Unnamed	Unknown	Beta-1,3 glucanase, GA2oxs, cystatin, cysteine protease, pectinesterase, TA29, and actin	Meiosis, tapetum development, and cell-wall formation/degradation	Omidvar et al. (2017)

## Molecular mechanisms of EGMS

### P/TGMS mechanisms at the RNA level

#### Non-coding RNAs

**Figure 3:**
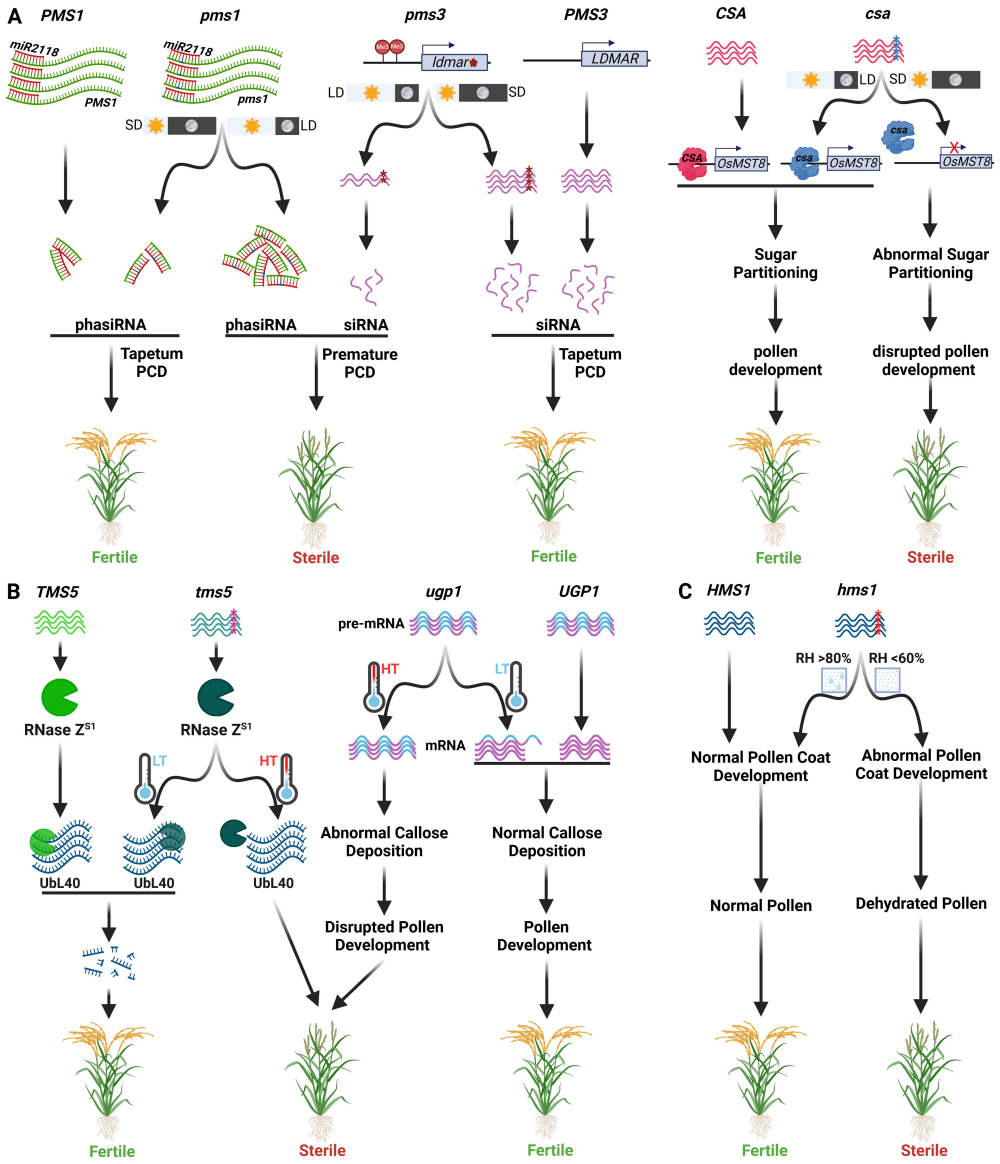
Molecular mechanisms of various EGMS systems used in two-line hybrid breeding. **(A)** Molecular mechanism of Photosensitive GMS (PGMS) involving male sterility genes pms1, pms3 and csa. Under long-day conditions, an SNP in the pms1 leads to differential expression of phasiRNAs, leading to male sterility (left). An SNP in the siRNA gene ldmar/pms3 leads to methylation of its promoter region under long-day conditions, thereby reducing siRNA production, which in turn leads to male sterility (middle). Under short days, a csa mutant (a transcription factor) leads to reduced OsMST8 expression, causing abnormal sugar partition and thereby disrupting pollen development (right). **(B)** Molecular mechanism of thermo-sensitive GMS (TGMS) caused by tms5 and ugp1 genes. An SNP mutation in the tms5 gene coding for an RNase ZS1 disrupts the processing of its cognate UbL40 mRNAs under high-temperature conditions, leading to male sterile phenotype (left). UGP1 gene is involved in callose deposition during pollen development. ugp1 mutant plants are male sterile at high temperatures due to failed processing of primary transcript containing introns. They become male fertile under low temperatures where primary transcripts are processed to remove introns (middle). **(C)** Molecular mechanism of humidity-sensitive GMS (HGMS) involving the male sterility gene hms1. The hms1 mutant shows abnormal pollen coat development at low humid conditions, leading to male sterility phenotype, which can be rescued at high humid conditions.

Non-coding RNAs (ncRNAs) play a pivotal role in governing P/TGMS in rice. The first PGMS rice line, NK58S, has a highly complex genetic mechanism. Both photoperiod and temperature act in a complementary manner to induce male sterility in NK58S line. Male sterility expression in NKS58S requires >13.75 h day length and >29°C temperature. In contrast, in *indica* background, PA64S, male sterility is predominantly governed by temperature which highlights the role of genetic background on expression of EGMS (Zhou et al., 2012; Fan and Zhang, 2018). PGMS in NK58S is governed by *pms1* and *pms3* (Zhang et al., 1994; Mei et al., 1999). *pms1* is a semi-dominant allele that codes for a long non-coding RNA (lncRNA) named *PMS1T* (**Figure 3A**). Under long-day conditions, *PMS1* transcript is targeted by miR2118, leading to the generation of 21-nt phased secondary small interfering RNAs (phasiRNAs). A single nucleotide mutation G to A in *pms1* near the miR2118 recognition site alters the secondary structure of *pms1* transcript, leading to a decrease in the accumulation of phasiRNAs. Thus, miR2118 seems to effectively cleave PMS1T under long daylight, increasing the production of 21-nt phasiRNAs. These phasiRNAs, in turn, target downstream anther-related genes leading to pre-mature programmed cell death of the tapetum (Fan et al., 2016).

The *PMS3* locus also encodes a lncRNA, Long-day-specific male-fertility-associated RNA (LDMAR) (Ding et al., 2012a; Ding et al., 2012b). The *pms3* allele has a single nucleotide substitution C to G. Under long-day conditions, effective pollen development requires elevated LDMAR expression. However, under long-day conditions, the single nucleotide mutation in *pms3* alters the secondary structure of LDMAR. This in turn increases LDMAR promoter methylation, consequently decreasing the production of LDMAR and causing pollen abortion (**Figure 3A**) (Ding et al., 2012b). Another gene *p/tms12* gene is also present at the *PMS3* locus. The P/TMS12-1 encodes a lncRNA, which generates a 21 nt small RNA named osa-smR5864w. A single nucleotide substitution in *p/tms12* leads to TGMS and PGMS in *indica* and *japonica* rice, respectively (Zhou et al., 2012). These discoveries underscore the role of the non-coding RNA in modulating PGMS/TGMS in rice. Despite cloning and examining their molecular mechanisms, how *pms1* and *pms3* (*p/tms121*) govern pollen mother cell and tapetum growth and their potential genetic interplay in *indica* and *japonica* lines remain to be elucidated.

Besides the lncRNA, 24-nt phasiRNAs were also identified to regulate P/TGMS. The monocot-specific *DICER-LIKE 5* (*DCL5*) is involved in generating 24-nt phasiRNAs. In maize, the *dcl5* mutants show differential accumulation of the 24 nt phasiRNAs in tapetal cells under high- and low-temperature environments. At temperatures ≥28°C, *dcl5* mutants show delayed/arrested tapetum development, leading to abnormal meiosis, which results in pollen abortion. However, at temperatures ≤26°C, *dcl5* mutants are partially or entirely fertile (Teng et al., 2020). Moreover, anther wall specific *ARGONAUTE 1d* (*AGO1d*) mediates phasiRNA biosynthesis and function by interacting with miR2118, miR2275, and miR2118-triggered 21-nt phasiRNAs through a 5′ uridine. In rice, the *ago1d* mutants show reduced levels of 21- and 24-nt phasiRNAs and rTGMS phenotype. At ~22°C, *ago1d* mutants show faulty tapetal cell PCD and meiosis, which leads to male sterility. However, at ~28°C, *ago1d* mutants could restore fertility (Shi et al., 2022; Si et al., 2023). Similarly, in maize, *MALE ASSOCIATED ARGONAUTE-1* and *-2* (*MAGO1* and *MAGO2*) are associated with 21-nt heat-activated phasiRNA (Hphasi), which are vital for normal pollen development under heat stress. To heat response, a large number of 21-nt Hphasi RNAs are accumulated in the wild type in comparison to RNA interference lines of *MAGO1* and *MAGO2*, causing male fertility at low temperatures (28°C/25°C day/night) and sterility under heat stress (35°C/25°C day/night) in RNAi lines (Lee et al., 2021).

#### Transcriptional regulation

A network of transcription factors operating at different stages control anther development. Several conserved transcription factors across plant species have been shown to be involved in P/TGMS. TGMS trait in commercially used rice lines Hengnong S‐1 and Tian1S is caused by alleles *tms9-1* and *OsMS1^wenmin1^*, respectively. The candidate gene identified for *tms9-1* is *OsMS1* (Qi et al., 2014). The *OsMS1* is a PHD-finger transcription factor that contains the nuclear localization signal, LXXLL motif (Qi et al., 2014; Wu et al., 2022). The T‐to‐C substitution within the LXXLL motif of the *OsMS1^wenmin1^* leads to Leu‐to‐Pro amino acid change that confers the TGMS trait (Wu et al., 2022). As a consequence, the mutant protein OsMS1^wenmin1^ is distributed in both the nucleus and cytoplasm, unlike the OsMS1 protein, which is restricted to the nucleus, thereby causing TGMS. At high temperatures (30°C), OsMS1^wenmin1^ is less abundant than OsMS1 in the nucleus. Further, OsMS1 and OsMS1^wenmin1^ proteins can bind in a temperature-dependent manner to the promoter of the *ETERNAL TAPETUM 1* (*EAT1*) gene and by interacting with TAPETUM DEGENERATION RETARDATION (TDR) regulate downstream genes (Wu et al., 2022). In soybean, a spontaneous mutation in *MS3*, an *Ostms9-1*/*AtMS1* ortholog, has intriguingly led to reverse PGMS, expressing male sterility under short days and fertility restoration under long days (Hou et al., 2022). Similarly, overexpression of the barley ortholog *HvMS1* exhibits a reverse TGMS, being sterile at <15°C and fertile at >20°C (Fernandez-Gomez et al., 2020). However, mutant alleles of *ms1* that lack the LXXLL motif and PHD domain exhibit male sterility independent of temperature and photoperiod in diverse plant species (Ito et al., 2007; Li et al., 2011; Fernandez Gomez and Wilson, 2014; Yang et al., 2019; An et al., 2020). These studies clearly demonstrate the conserved functions of these genes across monocot and dicot species and highlight the role of LXXLL and PHD domain in temperature/photoperiod sensitive male sterility/fertility. This also points to opportunity to engineer similar male sterility in other species.

In rice, the *carbon-starved anther* (*CSA*) and its paralog *CSA2* encoding, respectively, R2R3 MYB and MYB transcription factors control sugar partition during development of male reproductive organs in response to photoperiod (Zhang et al., 2010, Zhang et al., 2013; Wang et al., 2021). Mutations in either of these genes display PGMS. The *csa* mutant, a reverse PGMS line, exhibits male sterility during short days and male fertility during long days (**Figure 3A**) (Zhang et al., 2010; Zhang et al., 2013). In contrast, *csa2* mutant displays partial sterility during extended daylight periods and complete fertility under shorter day period (Wang et al., 2021). Besides this, under long-day conditions, the *csa-csa2* double mutant exhibits semi-sterility, suggesting dominant epistasis of *csa2*. Under short-day conditions, the double mutant is entirely sterile, mirroring the characteristics of *csa*. The *CSA* exerts direct regulation over the monosaccharide transporter gene *OsMST8*, an essential gene in the apoplastic sugar transport pathway, whereas *CSA2* exerts indirect regulation over *OsMST8* (Zhang et al., 2010; Wang et al., 2021). In short-day scenario, the *csa* mutant experiences a notable reduction in the expression of *OsMST8*, coupled with impaired carbohydrate transport from flag leaves to anthers, culminating in male sterility. Whereas during long-day conditions, *csa2* mutants exhibit suboptimal sugar transport from flag leaves to anthers, resulting in partial male sterility (Zhang et al., 2010; Wang et al., 2021). These outcomes underscore the shared molecular functions of CSA and CSA2 in influencing pollen development through orchestrated regulation of sugar transport from leaves to anthers, responsive to varying photoperiods.

#### Other RNA metabolism mechanisms

Temperature-dependent mRNA splicing and degradation have also been identified as molecular mechanisms controlling TGMS. The AnnongS‐1 (AnS‐1) is the pioneer *indica* line that shows male fertility at <24°C and male sterility at >26°C. A single recessive gene, *tms5*, governs this TGMS trait. The *TMS5* gene encodes RNase Z^S1^, which cleaves three *ubiquitin-ribosomal L40* (*UbL40*) mRNAs in the pollen mother cell. A nucleotide substitution from C to A at 71^st^ position in the *tms5* gene leads to male sterility at high temperatures due to the lack of function of RNase Z^S1^, leading to excessive accumulation of UbL40 mRNAs. However, under permissive temperature, RNase Z^S1^ is able to process the UbL40 mRNAs like in the *TMS5* line, so its pollen fertility is normal (**Figure 3B**) (Zhou et al., 2014). Further, *UDP-Glucose Pyrophosphorylase1* (*Ugp1*) expresses during anther development and catalyzes the production of UDP-glucose and pyrophosphate. The Ugp1-silenced, or -overexpression rice plants are male sterile. The *Ugp1* overexpressing plants are male sterile because of the impaired splicing of endogenous *Ugp1* primary mRNA. However, these plants revert to fertility at low temperatures (≤21°C) and under short day conditions (<12.5 h) due to more efficient splicing of *Ugp1* primary mRNA (**Figure 3B**) (Chen et al., 2007).

### P/TGMS mechanism at the protein level

#### TGMS through regulation of signal transduction

Rice TGMS gene *OsTMS15* encodes a Leucine‐rich repeat receptor‐like kinase (LRR–RLK) protein named MULTIPLE SPOROCYTE1 (MSP1). The TIR motif in the LRR region of MSP1 protein interacts with its ligand OsTDL1A to initiate tapetum development to support pollen formation. However, a point mutation in the TIR motif leads to TGMS phenotype in the *ostms15* mutant. At temperatures >29°C, the interaction of MSP1 and OsTDL1A proteins is reduced, causing male sterility. Whereas at lower temperatures <23°C, slow developmental rate and partial recovery of MSP1 and OsTDL1A interaction restore fertility to *ostms15* mutant (Yang et al., 2016; Han et al., 2023). Similar to *OsTMS15*, another two LRR–RLK proteins TMS10, and its homolog TMS10L, control pollen and postmeiotic tapetum development. The kinase activity of TMS10 plays a crucial role at higher temperatures in maintaining pollen viability. The *tms10* mutants exhibit male sterility at high temperatures (25-32°C) and male fertility at low temperatures (22-24°C) (Yu et al., 2017). Conversely, the *tms10l* mutant displays normal fertility at both high and low temperatures. However, double mutants (*tms10 tms10l*) exhibit male sterility at both temperatures, suggesting their functional redundancy in pollen development under low temperatures (Yu et al., 2017).

#### EGMS through altered pollen wall synthesis

The pollen wall is the most complex of any plant cell wall, protecting the pollen from damage and desiccation during its dispersal from anther to stigma (Blackmore et al., 2007; Xue et al., 2018). The pollen wall contains the inner intine and the outer exine layers with the exine coated with sporopollenin, a major biopolymer containing very-long-chain fatty acids (VLCFAs). Disruption of genes involved in sporopollenin synthesis leads to TGMS phenotype. For example, the rice *OsTMS18* gene encoding a glucose-methanol-choline (GMC) oxidoreductase is required pollen wall formation. Further, *OsTMS18* is regulated by tapetal transcription factor *OsMS188* (Zhang et al., 2022b). Whereas the upstream tapetum development regulator OsTDR regulates the OSMS188 through direct interaction with it (Han et al., 2021). A point mutation in *ostms18* leading to Gly to Ser amino acid change, confers TGMS phenotype. The *ostms18* mutant exhibits defective pollen exine wall at high temperatures (≥ 28°C). Under low temperatures (≤ 24°C), pollen double exine layer forms, leading to viable pollen and eventually fertility restoration (Zhang et al., 2022b). While the knocked out *osms188* lines exhibit altered PCD mechanism of the tapetum layer, defective anther cuticle and also absence of the sexine layer cause complete male sterility (Han et al., 2021). Similarly, a wheat rTGMS line BS366 is fertile at ≥20°C, and exposure to ≤10°C for five days at the PMC stage leads to male sterility (Tang et al., 2011). A *TaSCULP1* gene is expressed during sporopollenin assembly and plays a crucial role in sporopollenin *p*-coumaroylation and maturing exine. The overexpression of the *TaSCULP1* gene in the BS366 line restores the integrity of exine and fertility at low temperatures (Xu et al., 2023).

Pollination process begins with the adhesion of pollen exine to the stigma, followed by hydration and germination of the pollen grain (Wheeler et al., 2001). Therefore, mutations in genes involved in pollen adhesion and hydration can cause male sterility. Further, in some cases, high humidity conditions can rescue the male fertility, generating HGMS lines. For example, rice *OsOSC12* encodes a grass-species-specific triterpene synthase that catalyzes the cyclization of 2,3-oxidosqualene into poaceatapetol (Xue et al., 2018). Pollen grains of *ososc12* mutant at ambient or low humid conditions (<60%) show rapid dehydration, leading to male sterility. This desiccation is reduced at high humidity (>80%) thereby make pollen fertile. The male sterility is attributed to reduced synthesis of C16 and C18 fatty acids, triterpene esters and sterols, leading to ineffective adhesion and hydration (Xue et al., 2018). Similarly, OsCER1/OsGL1-4 synthesizes the C25 and C27 long-chain alkanes. The *oscer1*/*osgl1-4* mutants anthers show defective adhesion and hydration leading to male sterility under ambient humidity conditions. In contrast, high humid conditions rescue male fertility by increasing pollen adhesion and hydration (Yu et al., 2019). The *OsCER2*/*OsHMS1* encoding a β-ketoacyl CoA synthase interacts with HMS1I and catalyzes the biosynthesis of C26 and C28 VLCFAs, eventually helps in preventing pollen dehydration. Pollen produced on *oshms1* plants show impaired pollen grain adherence, hydration, and germination on the stigma under low humid conditions (<60% RH), leading to male sterility, which could be rescued at higher humid conditions (>80% RH)(**Figure 3C**) (Chen et al., 2020). Similarly, mutations in Arabidopsis genes *CER1* (Aarts et al., 1995), *CER2*, *CER2-LIKE* (Haslam et al., 2015; Zhan et al., 2018), *CER3*, *CER6*, *CER8*, and *CER10* (Koornneef et al., 1989) also induce HGMS under different humid conditions.

### Slow pollen development rate restores fertility

Recently, a slow pollen development rate under low temperature, short photoperiod, or low light intensity is identified as a general mechanism of male fertility restoration in P/TGMS lines. The slow pollen development rate was first identified in the Arabidopsis EMS mutant line *reversible male sterile* (*rvms*), which is male fertile at 17°C and sterile at 24°C. The *RVMS* gene encodes a GDSL lipase/hydrolase protein, and an SNP mutation/T-DNA insertion led to the TGMS phenotype, due to cytoplasmic leakage and defective nexine development. Zhu et al. (2020) identified that decrease in the microspore development rate might be the primary reason for the development of functional pollen at low temperatures. Further, when a slow microspore development rate mutant, *restorer of rvms 1* (*res1*), a weak allele encoding *A-TYPE CYCLIN-DEPENDENT KINASE;1* (*CDKA;1*), is crossed with *rvms* mutant, the generated double mutant is fertile at 24°C, confirming the slow growth rate of pollen rescues the pollen fertility. Shi et al. (2021) identified *res2* (or *qrt3*) as an additional mutant that can rescue *rvms* mutation. It encodes a polygalacturonase and exhibits delayed tetrad pectin wall degradation. Double mutants *res2 rvms-2* were fertile and displayed delay in degradation of the tetrad pectin wall (Shi et al., 2021). Similarly, the *res3*, encoding *UPEX1*, is also able to restore the fertility of the *rvms-2* TGMS line by delaying the degradation of tetrad callose wall and callose A6 secretion from tapetum to locule (Wang et al., 2022).

Further, *res1*, *res2* and *res3* alleles are also able to rescue male sterility of other TGMS mutants, such as *abcg26*, *acos5*, *cals5*, *cyp703a2*, and *rpg1*, which are sterile at 24°C and fertile at 17°C (Zhu et al., 2020; Shi et al., 2021). The *rpg1* is defective in primeexine development and is partially restored under low temperatures. However, the fertility restoration in the double mutant *rpg1 rpg2* is significantly reduced at low temperatures compared to the *rpg1* mutant, indicating the gene redundancy function plays a vital role in the fertility restoration of TGMS lines (Zhang et al., 2022a). Further, although these mutants *acos5*, *cals5*, *cyp703a2*, *npu* and rvms have been identified as TGMS lines, they also exhibit PGMS features, i.e., sterile at 16 h LD and fertile at 8 h SD (Zhang et al., 2020a). The fertility of these mutants has been restored at the SD or low-intensity light conditions due to slow development (Zhang et al., 2020a; Zhu et al., 2020; Shi et al., 2021). Besides Arabidopsis, the rice *OsTMS15* male sterility can be rescued by growing at low temperature which indicates that slow developmental rate at low temperatures recovers the OsTMS15 protein interaction with its ligand, thereby restoring pollen fertility (Han et al., 2023). Likewise, *B. napus* rTGMS lines 9012AB and male-sterility Lembke (MSL) share a similar male sterile chloroplast localized protein, BnChimera. BnChimera interacts with its fertility restorer BnaC9-Tic40, which restores the pollen fertility at high temperatures by slowing the flower development and synthesis of cell wall precursors (Schuhmann et al., 2022). These studies indicate that slow pollen development is a general mechanism to restore the fertility of P/TGMS lines to rectify pollen developmental defects.

### NGMS in plants

Among all plant nutrients, nitrogen constitutes 1-5% of the dry matter of plant tissues, and is integral to growth, metabolism and physiology, and also plays a vital role in various developmental processes (Muratore et al., 2021). In rice, Yang et al. (2022) recently identified a mutant defective in the gene encoding *electron transfer flavoprotein subunit β* (*etfβ*). This mutant exhibited both male and female sterility under N-starvation condition due to failure of sporogenous cells to enter into meiosis. Under N-deficient conditions, *ETFβ* helps in N reutilization and remobilization through its role in the catabolism of branched-chain amino acids (BCAA), and thereby helps meet the N requirements of spikelets, ensuring sexual reproduction. The *etfβ* mutant, under N-starvation conditions, exhibits excessive BCAA accumulation and reduced total N content, leading to male sterility. However, the *etfβ* mutant meiotic disorders can be rescued by exogenous N supply, thereby allowing the recovery of male fertility (Yang et al., 2022). As *etfβ* mutation causes both male and female sterility under N-deficient condition, this NGMS system cannot be used for hybrid seed production.

Although many of the genes described have not yet been put to practical use, these examples show how basic studies on pollen development can help us in identifying candidate genes and necessary mutations therein that can be harnessed to engineer EGMS in new crop species.

## Biotechnology-based male sterile systems

EGMS systems have been mostly restricted to rice. Although many GMS mutants have been identified in several crop species, their utility in commercial seed production is restricted due to issues such as unstable sterility or unintended side effects, the complexity of producing pure male sterile progenies on a large scale, labor-intensive and time-consuming plant breeding methods to transfer recessive male sterility traits into elite lines. Persistent challenges, like false selection and linkage drag appear unavoidable (Zhou et al., 2023). The advent of genetic engineering offered alternative ways to quickly generate male steriles in elite varieties in a single generation avoiding linkage drag (Gao, 2021).

The first transgenic male sterility/fertility system was developed in tobacco and rape seed through tapetum-specific expression of *barnase* and *barstar* genes sourced from *Bacillus amyloliquefaciens* strain H (IAM1521) (Mariani et al., 1990, Mariani et al., 1992). *barnase* gene codes for an extracellular ribonuclease enzyme with two cysteine residues, which is explicitly inhibited by a small 89 amino acids intracellular protein coded by the *barstar* gene. Male sterile plants were obtained by anther specific expression of the *barnase* gene (Mariani et al., 1990) whereas fertility restoration was achieved by anther-specific expression of the *barstar* gene (Mariani et al., 1992). A *bar* gene conferring tolerance to glufosinate herbicide was linked to *barnase* and *barstar* genes. Barnase-barstar system has been demonstrated to work in several crop species like rapeseed, Indian mustard, soybean, rice, vegetable species (Jagannath et al., 2002; Colombo and Galmarini, 2017). This system is comparable to CMS system and involves three lines for hybrid seed production. Kriete et al. (1996) engineered conditional male sterility system by anther-specific expression of a bacterial gene *argE* coding for *N*-acetyl L-ornithine deacetylase. When transgenic tobacco plants are sprayed with non-phytotoxic herbicide conjugate N-acetyl phosphinothricin, expression of *argE* gene in anthers produces toxic phosphinothricin in anthers causing their destruction. This conditional male sterility system is a two-line system. Ruiz and Daniell (2005) engineered cytoplasmic male sterility through plastid transformation. Transplastomic tobacco plants carrying *phA* gene coding for ß-ketothiolase driven by *psbA* promoter showed complete male sterility. Male fertility restoration was achieved when such plants were grown under continuous illumination. However, a practical male fertility restoration system for transplastomic male sterility has not been devised so far. Subsequently, several transgenic male sterility-fertility restoration systems have been reported in model plants (Shukla et al., 2017; Gautam et al., 2023). So far, only the Barnase-barstar system has been commercialized in a few countries such as USA, Canada and Australia. Objections to genetically modified crops in Europe and many other countries have prevented commercialization of transgenic male sterility systems for hybrid seed production.

**Figure 4:**
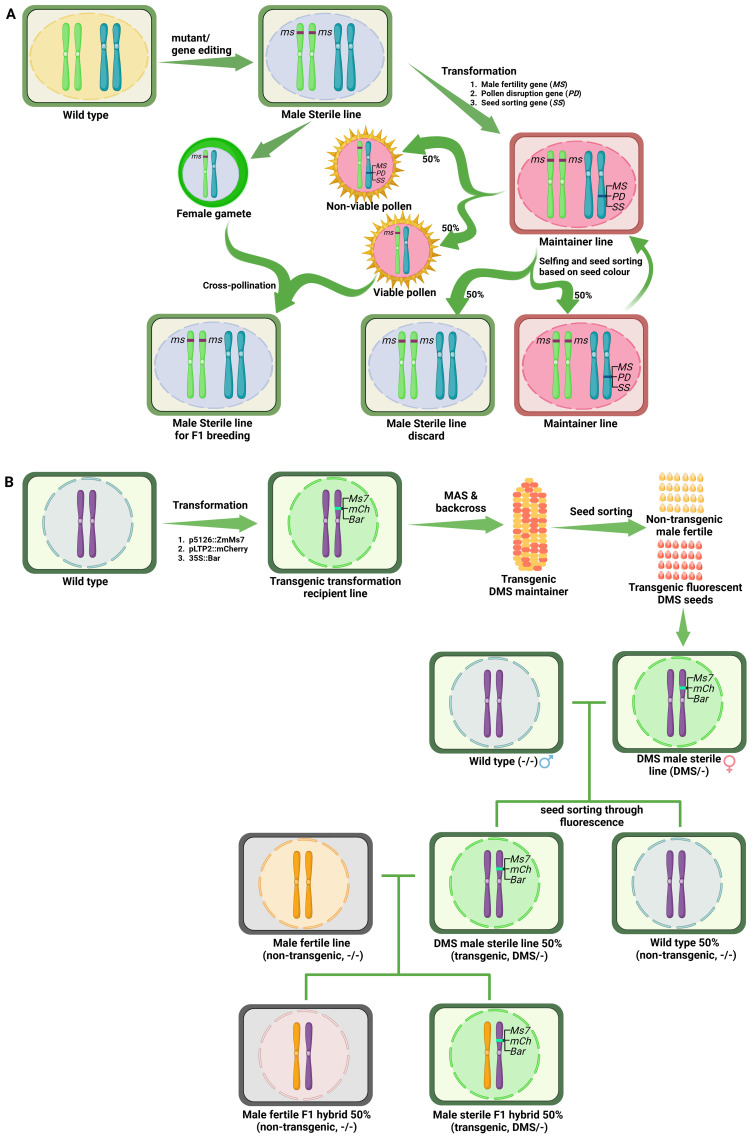
Examples of the third-generation hybrid seed production technologies. **(A)** Schematic diagram of DuPont-Pioneer propriety Seed Production Technology (SPT). This system uses the maintainer line developed from the male sterile line to produce non-transgenic hybrid seeds using transgenic technology. Male sterile lines with mutations in genes expressed in the sporophyte are obtained from natural mutants or through gene editing. The male fertile maintainer line is developed by transformation of the male sterile line with a triple gene construct containing i) the corresponding wild-type male fertility gene, ii) a pollen disruption gene operating at the gametophyte stage, and iii) a gene imparting seed color. The maintainer line produces only non-transgenic pollen because transgenic pollen is non-viable due to the presence of the pollen disruption gene. Therefore, a cross between the male sterile line and the maintainer line generates 100% male sterile progeny, which can be used for F1 hybrid seed production. Self-pollination of the maintainer line generates 50% maintainer line seeds and 50% male sterile seeds, which can be sorted through the seed-sorting marker. **(B)** Schematic diagram of dominant male sterility system (DMS). In this system, the transgenic DMS maintainer line is developed through transformation of triple gene construct containing i) tapetum specific dominant male sterility causing gene ZmMs7, ii) fluorescent marker gene mcherry, iii) herbicide tolerance bar gene, into the wild type plants. Male sterile seeds can be maintained through a cross between transgenic male sterile line and a wild type generates 50% non-transgenic male fertile seeds and 50% transgenic fluorescent sterile seeds, which can be sorting by fluorescence and also through bar gene. A cross between transgenic male sterile line and a fertile non-transgenic combiner line generates hybrid seeds, in which 50% non-transgenic hybrid seeds can be sorted through fluorescence.

To bypass the use of transgenic lines in hybrid seed production, Perez-Prat and Van Lookeren Campagne (2002) proposed generation of transgenic male fertile maintainer lines for recessive GMS lines. Using this concept, DuPont Pioneer developed the Seed Production Technology (SPT) system in maize utilising *ms45* male sterility gene (Wu et al., 2016). This system consists of a transgenic hemizygous SPT maintainer line (in *ms45/ms45* background) and carries wild-type *Ms45* transgene linked to a chimeric α-amylase gene containing amyloplast targeting signal peptide to disrupt the pollen germination, and a fluorescent seed color marker gene (*DsRed2*) (**Figure 4A**). The wild-type *Ms45* allele in transgenic plant rescues male sterility, producing 50% non-transgenic viable pollen. The rest 50% transgenic pollen are rendered non-viable due to breakdown of starch by the action of α-amylase transgene. Therefore, the cross between male-sterile line and hemizygous SPT maintainer line yields only non-transgenic male-sterile seeds for use in hybrid seed production. Further, the SPT maintainer line could be propagated through self-pollination where 50% seeds containing the SPT construct can be separated from non-transgenic seeds by fluorescent seed color sorting (Wu et al., 2016).

The SPT system has many advantages, such as the availability of a broad range of germplasm for parental selection, an increase in hybrid seed purity and yield, and the elimination of public concerns about GM crops. However, the SPT constructs show pollen transmission ranging from 0.002% to 0.518% depending on the SPT construct, genetic background and transformation event (Wu et al., 2016). To reduce transgene escape, multicontrol sterility (MCS) system carrying two pollen disruption genes (*α-amylase* and *Dam*) has been developed (Zhang et al., 2018). Further, the MCS system also includes a herbicide-resistant gene (*Bar*) for selecting transgenic seeds (Zhang et al., 2018).

Similar to SPT and MCS systems, a nuclear male sterile rice maintainer line was developed by cloning *OsNP1*, *α-amylase* and *DsRed2* genes into the EMS mutant *osnp1* (Chang et al., 2016). Likewise, Du et al. (2020) created transgenic maintainer line in tomato by introducing the wild type fertility allele linked to seedling color marker in male sterile mutant. The offspring of homozygous male sterile plants and hemizygous maintainers produce 50% non-transgenic male sterile and 50% transgenic fertile seedlings, which can be sorted out through the seedling color before transplantation and obtain a large quantity of recessive genic male-sterile plants.

The above SPT systems are based on recessive male sterility and require prior knowledge of male sterility causing genes and male sterile mutants. If the mutants are not available, two different stocks have to be generated through transformation; the recessive male sterile line through genome editing (followed by selection of transgene-free lines) and transgenic male fertile maintainer line. To circumvent this major bottleneck, An et al. (2020) developed a dominant male sterility system (DMS) in maize, which would require development of only one transgenic line for hybrid seed production. They demonstrated that premature expression of a tapetum specific gene *ZmMs7* causes dominant male sterility through delayed PCD of tapetum leading to defects in pollen exine deposition. By linking male sterility construct (*p5126::ZmMs7*) with the fluorescent marker gene *mcherry* (expressed in aleurone layer of seed) and constitutively expressed *bar* gene (conferring Basta herbicide tolerance), they developed a system to track the male sterile individuals at the seed stage. The dominant male sterility-based hybrid seed production system is depicted in **Figure 4B**. The male sterile line could be multiplied by crossing with its non-transgenic counterpart, which will yield 50% transgenic (male sterile) seeds that could be sorted by fluorescence screening. The presence of *bar* gene in male sterile lines provides additional opportunity to eliminate any non-transgenic plants in hybrid seed production field. Similarly, hybrid seeds could be produced by crossing with non-transgenic combiner line where non-transgenic hybrid seeds could be separated for commercial use by fluorescence screening. The *Ms7* gene is highly conserved among diverse plant species such as rice, barley, Arabidopsis, Brassica etc. Further, transformation of Arabidopsis with the above construct gave dominant male sterility as observed in maize (An et al., 2020) thereby demonstrating that this is a versatile hybrid seed production system. Thus, DMS system provides a robust, widely applicable approach to engineer pollination control system across crop species.

**Figure 5:**
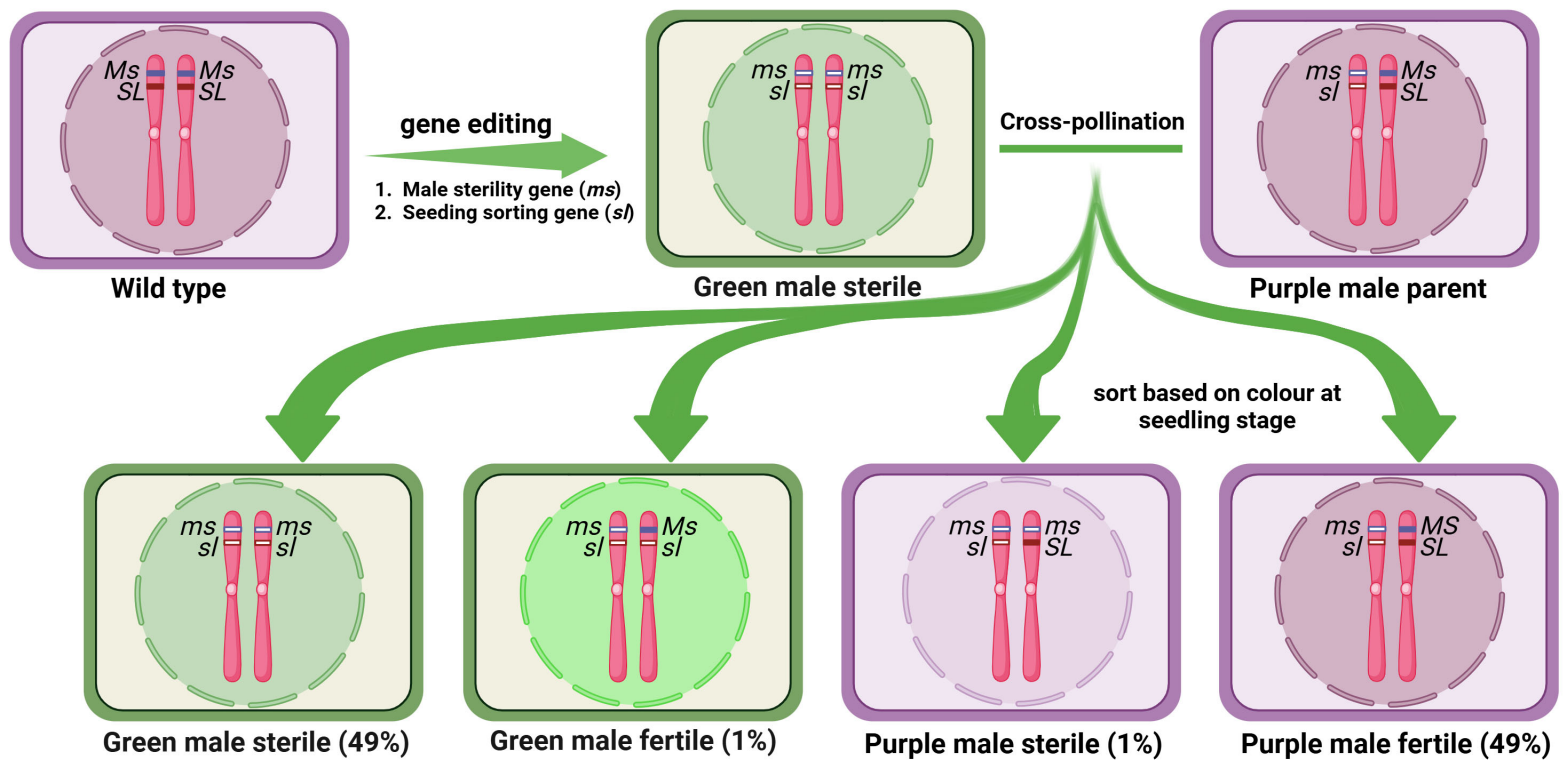
Schematic diagram of use of CRISPR/Cas double knockout mutant in hybrid seed production. Here, a male sterile line developed through the knockout of two closely linked genes, a male sterility-causing gene and a morphological seedling marker gene. A cross between the double knockout mutant and the wild type generates the 1:1 ratio of male sterile and male fertile progenies and a few recombinants, which can be sorted based on the color at the seedling stage.

In a further attempt to avoid the use of transgenics altogether, Zhou et al. (2023) identified a male sterility gene (*TM6*) in tomato which is closely linked to a seedling marker gene *Anthocyanin Without* (*AW*) coding for DFR enzyme. Male sterile TM6 mutants (ms-15, ms-26 and ms-47) can thus be identified at the seedling stage by hypocotyl color. They employed CRISPR/Cas9 system to knock out these two genes to obtain male sterile line lacking anthocyanin pigment in the seedling hypocotyls. This system exhibited male sterility along with a distinctive marker (green hypocotyls), facilitating the identification of sterile plants at the seedling stage. However, it still gives rise to a few recombinants between these linked genes which need physical rouging (**Figure 5**). Likewise, mutant alleles of tomato *MS-10* gene, *ms-10*, *ms-24*, *ms-35* and *ms-36*, cause male sterility and *MS-10* is linked to *anthocyanin absent* (*aa*) gene (Zhang et al., 2024). Thus *ms-10* and *aa* gene pair provides another opportunity to use linked seedling marker for selecting male sterile progenies. However, finding a closely linked morphological marker to the male sterility gene in each crop species will be a challenge.

## Conclusions and perspectives

EGMS has demonstrated significant value in hybrid seed production due to its ability to exploit environmental conditions to induce male sterility and fertility phenotype. However, the molecular mechanisms underlying EGMS systems have been extensively studied only in rice, leaving a major gap in understanding their mode of function in other major crops. Investigations into EGMS systems of other major crops are crucial to maximize their utility. Additionally, studies exploring the impact of climate change on EGMS systems are also needed to ensure their stability and adaptability in hybrid seed production. Meanwhile, SPT, MCS and DMS systems offer innovative solutions to overcome the challenges of conventional seed production methods such as manual emasculation and dependence on environmental conditions. Despite their advantages, these systems have limitations such as the need for sophisticated seed sorting machinery and increased cost of seed production associated with 50% unused seeds. Addressing these challenges through technological advancements and cost-effective solutions will be crucial for optimizing their utilization in hybrid seed production. Seedling marker linked male sterility as demonstrated in tomato (Zhou et al., 2023) is a very good approach. However, detecting and removing low frequency recombinant male fertile plants is a problem in seed production. In recent years gene editing technologies have been employed to create chromosomal inversions at target locations (Zhang et al., 2017; Schmidt et al., 2019; Ronspies et al., 2021; Hu et al., 2024). Thus, creating a small inversion between the seedling marker gene and male sterility gene can overcome the problem of recombination and thereby make this a perfect pollination control system.

Heterosis benefits have largely been restricted to a few major global crops. In particular, lack of reliable male sterility/fertility control systems has discouraged heterosis breeding in many crops such as pulses, crops bearing small, hermaphrodite flowers such as millets (e.g. finger millet, foxtail millet etc.), seed spices, amaranth. The accumulated knowledge of genes causing male sterility in crops, availability of whole genome sequences, conserved genes and genetic pathways of male gamete development offer great scope for developing male sterility systems in these crops. Although the third-generation DMS system presents the best promise, CMS and EGMS systems will continue to serve the hybrid seed industry, especially in countries where transgenics are not accepted.

Pollen competition is a phenomenon where pollen of different genetic constitutions display variation in the rate of pollen tube growth, leading to one type of pollen showing preferential transmission through the male side. For example, Arabidopsis *Cals5* gene mutants produce pollen whose tubes lack callose, a major component in the pollen wall. The *cals5-3* mutant produces seeds upon selfing (no pollen competition) but mutant pollen competes poorly against wild type pollen (Nishikawa et al., 2005). Hence, in heterozygous plants mutant allele is poorly transmitted from the male side. Similarly, in rice, loss-of-function mutants of pollen expressed hexokinase gene *Hxk5*, produce viable pollen and give 5-10% seed set upon selfing. However, *hkx5* mutant pollen grow slowly and fail to compete with wild type pollen. Hence, in heterozygous (*+/hkx5*) plants, the mutant allele is not transmitted from the male side (Lee et al., 2020). A similar slow pollen germination mutant has been identified in ragi (*Eleucine coracana*), which shows promise for heterosis breeding (Manjappa et al., 2015). Such slow pollen germination mutants offer unique opportunity for hybrid seed production because homozygous mutant stocks can serve as male sterile line for hybrid seed production and can also be multiplied through selfing. Considering that starch metabolism pathway in pollen grains is highly conserved, slow pollen germination feature is particularly attractive. In crops where seedlings are transplanted, genic male sterility combined with seedling/herbicide marker can be effectively used for heterosis breeding.
